# Problem Technology Use, Academic Performance, and School Connectedness among Adolescents

**DOI:** 10.3390/ijerph19042337

**Published:** 2022-02-18

**Authors:** Hugues Sampasa-Kanyinga, Hayley A. Hamilton, Gary S. Goldfield, Jean-Philippe Chaput

**Affiliations:** 1 School of Epidemiology and Public Health, University of Ottawa, Ottawa, ON K1G 5Z3, Canada; jpchaput@cheo.on.ca; 2 Healthy Active Living and Obesity Research Group, Children’s Hospital of Eastern Ontario Research Institute, Ottawa, ON K1H 8L1, Canada; ggoldfield@cheo.on.ca; 3 Institute for Mental Health Policy Research, Centre for Addiction and Mental Health, Toronto, ON M6J 1H4, Canada; hayley.hamilton@camh.ca; 4 Dalla Lana School of Public Health, University of Toronto, Toronto, ON M5T 3M7, Canada

**Keywords:** screen, smartphone, tablet, laptop, computer, school outcomes, school belonging, youth

## Abstract

Background: Little is known about the association between problem technology use in adolescents and school-related outcomes. The purpose of this study was to determine the prevalence of problem technology use and examine its association with academic performance and school connectedness in a sample of students across Ontario, Canada. Methods: Self-reported data from a sample of 4837 students in grades 9 to 12 (mean age: 15.9 years; 49.5% females) were cross-sectionally analyzed. Ordered logistic regression models were adjusted for important covariates. Results: We found that 35.8% of students used their screen device for at least 5 h a day and about 18.6% had moderate-to-serious symptoms of problem technology use, a prevalence that was higher in females (22.4%) than males (14.9%). Heavy technology use was differentially associated with lower academic performance and lower levels of school connectedness in males and females. Having moderate-to-serious symptoms of problem technology use was associated with lower academic performance among males (AOR = 0.68, 95% CI = 0.53–0.87) and females (AOR = 0.66, 95% CI = 0.52–0.84). It was also associated with less school connectedness in both males (AOR = 0.65, 95% CI = 0.50–0.86) and females (AOR = 0.63, 95% CI = 0.51–0.78). Conclusion: Excessive use and problem technology use are highly prevalent among secondary school students, and they are associated with lower academic performance and lower levels of school connectedness.

## 1. Introduction

Technology use has become the most common leisure-time activity for many adolescents around the world [1]. Technology use refers to screen-based activities related to computers, laptops, tablets, smartphones, or gaming consoles for example. A recent report indicates that about 35 percent of secondary school students spend five or more hours daily on technology use in their free time, a five percent increase from 2017 (29.5%) [2]. An additional 34 percent of students spend three to four hours per day using technology [2]. These statistics are concerning because screen time behaviours can extend into adulthood [3], and they are associated with increased morbidity and mortality [4,5]. For optimal health benefits, the Canadian 24 h movement guidelines for youth aged 5 to 17 years recommend spending no more than 2 h per day in recreational screen time [6]. Technology use can be associated with numerous benefits for child development; for example, research has shown that it can improve cognitive function and enhance the ability to work, socialize, and/or spend leisure time [7]. However, a vast majority of adolescents exceed the recreational screen time guidelines. Research indicates that heavy technology use is associated with lower academic performance among adolescents [8,9]. It is not clear, however, if heavy technology use is associated with lower school connectedness in large-scale and representative data of adolescents. Previous work [10] showed that heavy social media use is negatively associated with school connectedness and academic performance in a sample of more than 10,000 middle and high school students obtained from the 2013 cycle of the Ontario Student Drug Use and Health Survey (OSDUHS). Contrary to social media use, technology use is a more global measure, encompassing different types of activities, such as internet use, instant messaging, social media use, and computer gaming. Excessive technology use has also been associated with several adverse health outcomes, such as mental health problems, poor sleep, injuries, aggressive behaviours, and addiction-related issues [11,12,13].

Problem technology use is an emerging issue that parents, teachers, and health professionals are concerned about [14,15]. It is a behavioural problem defined by being overly worried about technology use, driven by an uncontrollable urge to use it, and devoting so much time and effort to technology that it impairs other important life areas [16]. Several broad terms have been used to describe problem technology use in the literature, including but not limited to internet addiction disorder, problem internet use, pathological internet use, technology-based addiction, excessive technology use, pathological technology use, problem electronic media use, and digital dependency. To date, there is no formal diagnosis in the Diagnostic and Statistical Manual of Mental Disorders 5th edition (DSM-5) or in the International Classification of Diseases 11th Revision (ICD-11) related to the broader issue of problem technology use, but this typology as a behavioural addiction is under consideration in the next iteration of the DSM [17]. However, several validated screening tools are available to probe potential signs of problem technology use, such as the Problem Internet Use Questionnaire [18]. This questionnaire is a self-administered tool that assesses harms such as preoccupation with online use, neglect of non-online activities, and inability to stop using the internet [16].

Existing research examining sex differences in problem technology use among adolescents has not been conclusive. Some studies have found that females are more likely than males to report signs of problem technology use. For example, Liu et al. [19] found that females were more likely than males to endorse the subjective measures of problematic internet use in a sample of more than 3500 United States high school students. Similarly, Mihara et al. [20] found that females exhibited more problematic internet use than males using a sample of junior and senior high school students. However, other investigations found that males have more problematic internet use than females [21,22,23], whereas others have found no such differences [24]. Regardless, previous studies using the OSDHUS data have shown that females are more likely than males to use electronic media and use them for a longer duration [25,26]. Research has also indicated that females spend more time using smartphones and social media networks, while males spend more time in online gaming [24,27].

Research has suggested that health anxiety and fear of missing out are important factors explaining problem use among heavy technology users [28,29]. There is also growing evidence on the associations between technology use during leisure time and adverse health-related outcomes [8,30]. Problem technology use can result in worrisome effects on the life of adolescents, their family, and the entire community [15]. However, less is known about the association of problem technology use with academic performance and school connectedness at the population level of adolescents. Given the substantial spread of technology use in the daily life of many adolescents, particularly in the current context of the COVID-19 pandemic [14], it is crucial to understand the impact of problem technology use on academic performance and school connectedness among adolescents. Thus, the primary objectives of this study were to determine the prevalence of problem technology use and to examine its association with academic performance and school connectedness in a sample of secondary school students in Ontario, Canada. The secondary objectives were to examine the association of high technology use with academic performance and school connectedness, and test if all the associations vary between female and male adolescents. It is hypothesized that problem technology use and high technology use would result in lower school performance and lower levels of school connectedness among secondary school students. It is also expected that the associations would be stronger among females than males.

## 2. Materials and Methods

The present report is in accordance with the Strengthening the Reporting of Observational Studies in Epidemiology (STROBE) guidelines for observational cross-sectional studies [31,32].

### 2.1. Design

The OSDUHS is an Ontario-wide survey that is conducted every 2 years among students in grades 7–12 who are enrolled in the Ontario publicly funded school system (English language public, English language Catholic, French language public, and French language Catholic) [33]. Students excluded from the survey’s target population (out-of-scope) were those enrolled in private schools, those who were home-schooled, those institutionalized for correctional or health reasons, those schooled in First Nation communities, on military bases, or in the remote northern region of Ontario. These exclusions represent a small proportion of the Ontario student population (about 8%). The OSDUHS is a self-administered, anonymous survey that monitors awareness and use of alcohol, tobacco, other drugs, and the mental and physical well-being of Ontario students. The 2019 OSDUHS protocol was approved by the Research Ethics Boards at the Centre for Addiction and Mental Health and York University, as well as 34 school board research review committees. Active parental consent and student assent were required for all participating students. The OSDUHS uses a two-stage cluster sample design involving a random selection of schools and classes stratified by region and school type (i.e., middle vs. secondary). Within each strata, schools were selected with probability-proportional-to-size by means of systematic selection without replacement (WOR), and within selected schools, classes were selected with equal probability and WOR.

### 2.2. Sample

Data for the 2019 OSDUHS were collected from November 2018 to June 2019 across 992 classes, 263 schools, and 47 school boards. A total of 14,142 middle and high school students completed the survey, with a student completion rate of 59%. Nonresponses were due to student absenteeism (12%) and unreturned consent forms or parental refusal (29%). Current cross-sectional analyses are restricted to the random half sample of secondary school students (*n* = 5273) who completed form A of the questionnaire that asked about technology use in their free time, and related problems. Detailed descriptions of the OSDUHS are available elsewhere [33].

### 2.3. Measures

Technology use was assessed using the following question: “About how many hours a day in your free time do you usually spend on electronic devices texting, messaging, emailing, chatting, watching videos, playing games, using social media (such as Instagram, Snapchat, Facebook), or surfing the internet?” Response options referred to daily use (less than 1 h a day, about 1 h a day, 2 h a day, 3 to 4 h a day, 5 to 6 h a day, 7 h or more a day); use these devices, but not daily; and do not use these devices. Based on the Canadian 24 h movement guidelines [6], response options related to daily use were combined to create 3 categories: daily use of technology of 2 h or less (recommended use, coded as 0), daily use of 3 to 4 h (moderate use, coded as 1), and daily use of technology of 5 h or more (heavy use, coded as 2).

Problem technology use was measured using the 6-item Short Problem Internet Use Test (SPIUT) [18]. The scale was adapted from the longer Compulsive Internet Use Scale, and it measures various dimensions, including preoccupation, loss of control, withdrawal, conflict with family/friends, and coping. The following six questions were asked: “How often do you find that you are staying on electronic devices longer than you intended?”, “How often do you neglect homework because you are spending more time on electronic devices?”, “How often are you criticized by your parents or your friends about how much time you spend on electronic devices?”, “How often do you lose sleep because you use electronic devices late at night?”, “How often do you feel nervous when you are not using electronic devices and feel relieved when you do go back to using them?”, and “How often do you choose to spend more time on electronic devices rather than go out with your friends?”. The response options for all six items ranged from “never” (coded as 0) to “very often” (coded as 4) and were summed to create a score ranging from 0 to 24. For analysis purposes, two problem technology use variables were constructed from this summated score: a moderate-to-serious problem with technology use (scores of 14 or higher) and a serious problem with technology use (scores of 19 or higher) [18]. Cronbach’s alpha for the present data was 0.78, indicating high internal reliability [34].

Academic performance was measured by the following question: “On average, what marks do you usually get in school?”. Response options ranged from A (coded as 1) to F (coded as 6): “A (80–100%)”, “B (70–79%)”, “C (60–69%)”, “D (50–59%)”, and “F (below 50%)”. Responses were reverse coded in our analyses such that higher values indicate higher academic performance. It was treated as a scale variable ranging from 1 to 6.

School connectedness was measured using the following three statements: “I feel close to people at this school”, “I feel like I am part of this school”, and “I feel safe in my school”. Response options ranged from “strongly agree” (coded as 1) to “strongly disagree” (coded as 4). They were reverse coded in our analyses and summed such that higher scores indicate higher levels of school connectedness. This composite measure is widely used among adolescents [35,36,37] and has been suggested to be a good measure of school connectedness among middle and secondary school students [38]. The index indicated acceptable internal consistency with a Cronbach’s alpha of 0.68 [34].

Covariates included in the analyses were age (in years), ethnoracial background, subjective socioeconomic status, tobacco cigarette smoking, alcohol consumption, and cannabis use. Subjective SES was assessed using an adapted version of the MacArthur Scale of Subjective Social Status [39,40]. The MacArthur Scale is a reliable measure of subjective social status [40]. Tobacco use was measured with an item that asked students how often they smoked cigarettes over the past 12 months. Alcohol use was measured with a question asking students how often they drank alcohol (liquor, wine, beer, coolers) over the past 12 months. Cannabis use was measured with a question asking students how often they used cannabis (e.g., “marijuana”) over the past 12 months. All three measures were treated as scale variables ranging from 1 to 9 for alcohol use, 1 to 10 for tobacco use, and 1 to 7 for cannabis use, with higher numbers reflecting greater use.

### 2.4. Statistical Analysis

All analyses were conducted using the survey procedure in Stata 15.1 to account for the complex survey design of the OSDUHS. Given that sex interactions between technology use and academic performance and between problem technology use and school connectedness were significant (*p* < 0.05), analyses were stratified by sex. We first described the sample using proportion, mean, standard deviation, and median. Then we investigated the prevalence of technology use and symptoms of problem technology use. Finally, we conducted univariable and multivariable ordered logistic regression analyses to examine the associations of time spent using technology and problem technology use (independent variables) with academic performance and school connectedness (dependent variables) among adolescent females and males. Tests indicated that the final models did not violate the proportional odds/parallel-lines assumption [41]. Models were adjusted for age, ethnoracial background, subjective socioeconomic status, tobacco cigarette smoking, alcohol consumption, and cannabis use. Covariates were selected based on their availability in the dataset and their associations with the dependent and independent variables. Age, ethnoracial background, and subjective socioeconomic status were included to account for the potential confounding effects of sociodemographic characteristics on the link between electronic media use and school outcomes in children and adolescents [10,42]. Tobacco cigarette smoking, alcohol consumption, and cannabis use were added to control for the confounding effects of substance use and its association with both school outcomes and addictive behaviour [43,44]. Results are expressed as odds ratios (OR) and their 95% confidence intervals (CI). A total of 4837 (92%) students had complete data in all variables included in our analyses. Individuals with missing data (*n* = 436) were more likely than those with completed information to be of other ethnoracial backgrounds, to report recommended daily technology use (i.e., no more than 2 h per day), and exhibit less problem technology use.

## 3. Results

The demographic characteristics of the sample are outlined in Table 1. About half of the sample were females (49.5%) and of white ethnoracial background (51.4%). Nearly 29% of students reported using technology for no more than 2 h per day. An additional 34.9% reported using technology for 3 to 4 h per day, and 35.8% reported using it for more than 5 h per day. Nineteen percent of students reported symptoms that may suggest moderate-to-serious problem technology use, and 2.9% reported symptoms that may indicate a serious problem with technology use. Females were more likely than males to report using technology for a longer duration and to report moderate-to-serious or serious symptoms of problem technology use. Females were also more likely to report higher academic performance but lower levels of school connectedness than their male counterparts.

**Figure 1:**
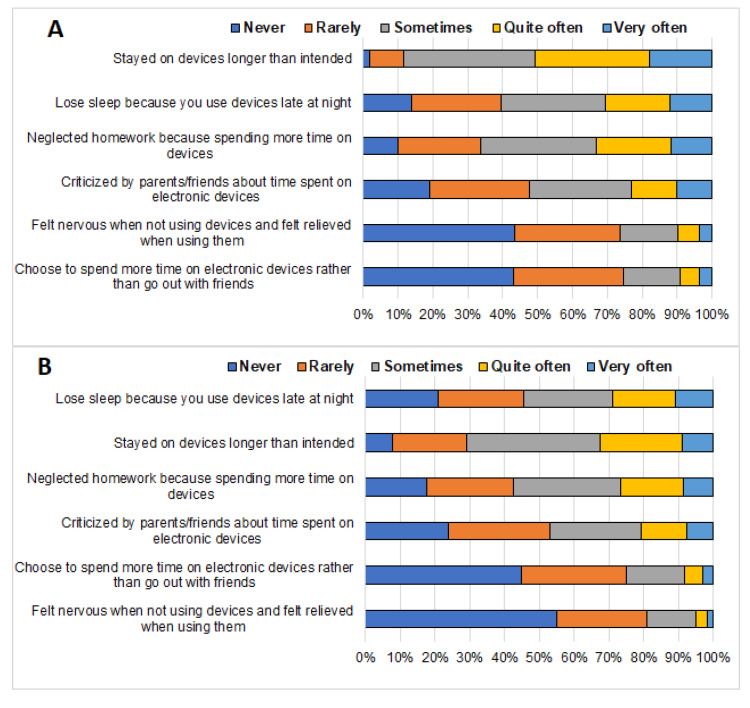
Symptoms of problem technology use among female (Panel **A**) and male (Panel **B**) adolescents.

Figure 1 displays responses regarding symptoms of problem technology use among adolescent females (Panel A) and males (Panel B). Based on students who indicated that they experienced given symptoms “very often”, the most prevalent symptom of problem use among females was staying on the device longer than intended, followed by losing sleep because you use devices late at night and neglecting homework because of spending more time on devices. However, the most prevalent symptom of problem technology use among males was “losing sleep because you use devices late at night”, followed by “staying on the device longer than intended” and “neglecting homework because of spending more time on devices”.

Results of ordered logistic regression analyses examining the associations of time spent using technology with academic performance and school connectedness among adolescent females and males are summarized in Table 2. Compared to technology use for 2 h or less per day, daily technology use of 3 to 4 h (AOR = 0.69, 95% CI = 0.52–0.91) or more than 5 h (AOR = 0.46, 95% CI = 0.34–0.61) was associated with lower academic performance among females, while only daily use of more than 5 h was associated with lower academic performance among males (AOR = 0.73, 95% CI = 0.55–0.98). Daily technology use of more than 5 h was also associated with lower levels of school connectedness in both males (AOR = 0.72, 95% CI = 0.56–0.92) and females (AOR = 0.75, 95% CI = 0.10–0.60).

Table 3 presents results from the ordered logistic regression analyses examining the associations of moderate-to-high symptoms of problem technology use with academic performance among adolescent females and males. Having moderate-to-serious symptoms of problem technology use was associated with lower academic performance among males (AOR = 0.68, 95% CI = 0.53–0.87) and females (AOR = 0.66, 95% CI = 0.52–0.84). The total score and individual symptoms of problem technology use were also associated with lower academic performance among males and females, with some exceptions. Staying on devices longer than intended was not associated with academic performance in both sexes, whereas getting criticized by parents or friends about time spent on electronic devices was associated with lower academic performance in females (AOR = 0.86, 95% CI = 0.78–0.94), but not males (AOR = 0.98, 95% CI = 0.91–1.05).

Results of ordered logistic regression analyses examining the associations of moderate-to-high symptoms of problem technology use with school connectedness among adolescent females and males are summarized in Table 4. Having moderate-to-serious symptoms of problem technology use was associated with lower levels of school connectedness among males (AOR = 0.65, 95% CI = 0.50−0.86) and females (AOR = 0.63, 95% CI = 0.51−0.78). The total score and individual symptoms of problem technology use were also associated with lower academic performance among males and females, with some exceptions. Staying on devices longer than intended was not associated with academic performance in both sexes, whereas losing sleep because of late-at-night device use was associated with lower academic performance in females (AOR = 0.90, 95% CI = 0.85−0.96), but not males (AOR = 0.97, 95% CI = 0.89−1.06).

## 4. Discussion

The results found in this study showed a high prevalence of excessive and problem technology use in a sample of secondary school students in Ontario, Canada. Findings further indicated that sex was a significant moderator of the associations of technology use with academic performance and school connectedness. Daily technology use of 3 to 4 h or more than 5 h were associated with lower academic performance among females, while only daily use of more than 5 h was associated with lower academic performance among males. Daily technology use of more than 5 h was also associated with lower levels of school connectedness in both males and females. Having moderate-to-serious symptoms of problem technology use was associated with lower academic performance and lower school connectedness in males and females. These findings underscore a clear need for intervention efforts that address technology use among secondary school students.

While research suggests that moderate technology use may have benefits for child development [45], it is also well known that heavy technology use is associated with poor school outcomes among children and adolescents [46,47,48,49]. Our results are consistent with previous studies indicating that heavy technology use (either as a global measure or specific technology use) is associated with negative academic performance and lower levels of school connectedness among adolescents [10,47,50]. These findings provide further support to the 24 h movement guidelines, which recommend limiting recreational screen time exposure to no more than 2 h per day [6]. Our results are also consistent with studies that have found that internet problem use is associated with negative academic performance and lower school connectedness among adolescents [51,52,53]. For example, Hayixibayi et al. [51] have found that problem internet use was associated with lower school connectedness in a sample of 6552 Chinese adolescents. They also found that these associations were stronger among older adolescents than their younger counterparts [51]. Technology use has greater mass appeal among adolescents, and its potentially addictive nature makes it more concerning [54]. Monitoring and reducing time spent on technology could be appropriate behavioural strategies to prevent problem technology use and possible means to improve school connectedness and academic performance among secondary school students.

Several mechanisms could explain the association of heavy technology use and problem technology use with academic performance and school connectedness. First, heavy technology use, particularly daytime use, could displace time devoted to learning activities, such as studying, reading, or doing homework, which could result in negative academic performance [55]. Second, heavy technology use, particularly late-night use, may displace sleep or shift circadian rhythms towards a later midpoint of sleep [6,7,16], leading to daytime sleepiness and fatigue, thus lowering the ability to optimally perform at school. Third, heavy technology use may constitute an essential source of stress and negative emotions due to direct exposure to stressful or harmful content, and unfavourable social comparisons that could undermine academic performance and school connectedness. Fourth, technology use may distract and displace time that adolescents allocate for schoolwork. Heavy technology use negatively impacts school connectedness, as students become disconnected from school and lose interest in school, which can lead to poorer academic performance. Research has shown that heavy social media use is associated with negative interpersonal relationships and lower social and school connectedness levels among adolescents [56]. Moreover, we have previously found that lower school connectedness could explain the link between heavy social media use and poor academic performance among middle and high school students [10]. Finally, health anxiety and fear of missing out have been identified as essential factors explaining problem use among heavy technology users [57].

We found that females were not only more likely than males to report using technology for a longer duration, but they were also more likely to develop problem technology use than their male counterparts. These findings are somewhat consistent with previous studies indicating such sex differences. For example, Liu et al. [19] found that females were more likely than males to endorse the subjective measures of problematic internet use in a sample of more than 3500 United States high school students. Similarly, Mihara et al. [20] found that females exhibited more problematic internet use than males using a sample of junior and senior high school students. However, other investigations found that males have more problematic internet use than females [21,22,23], whereas others have not found sex differences in problematic internet use among adolescents [24]. Inconsistent findings may be due, in part, to methodological differences, the continually evolving nature of technology use, and the variability and range of different types of technology use. It is well known that sex differences vary by specific technology use [27,58]. For example, females spend more time using smartphones and social media networks, while males spend more time in online gaming [24,27]. However, information on specific types of technology use was not captured in our survey. Nevertheless, the present study extends the evidence base by documenting sex differences in the association between heavy technology use and academic performance in adolescents. We found that daily technology use of 3 to 4 h or more than 5 h was associated with lower academic performance among females, while only daily use of more than 5 h was associated with lower academic performance among males. The observed differences are hard to explain, particularly with the continuously evolving nature of technology use. Males are more physically active and more engaged in school sports [59], and this could somewhat buffer the effects of moderate technology use. It is also possible that technology use of 3 to 4 h per day is not sufficiently intense to impact academic performance among males. Future research is needed to disentangle these differences.

This study has several strengths, including the use of a large and representative sample of adolescents, the use of survey procedures to accommodate the complex survey design, an adjustment for important covariates, and an examination of sex differences. Moreover, this study examined the associations for individual symptoms of problem technology use, total combined score, and categories, which provide a better understanding and more confidence of the observed associations. Finally, with research indicating the substantial spread of technology use in the life of many adolescents, particularly in the current context of the COVID-19 pandemic, this study is timely and will inform school health promotion efforts.

Several limitations should be considered when interpreting the results. First, this was a cross-sectional study; therefore, we cannot establish a causal relationship between problem technology use, academic performance, and school connectedness. Second, our data are based on self-reports; thus, there is a potential for recall and desirability biases. Third, the survey does not include adolescents who were absent or dropped out of schools, in whom symptoms of problem technology use could be typically elevated. Therefore, it is possible that the observed strength of associations herein is underestimated. Fourth, the present study did not examine the association between time spent on specific technology use (e.g., smartphones, tablets, laptops, computers, gaming consoles, etc.) with academic performance and school connectedness. Research has shown that types of technology use are differentially associated with academic performance among adolescents [47]. Thus, studies must capture the contributions of specific technology use modalities and their impact on academic performance and school connectedness among secondary school students. Identifying which types of technology use are most strongly associated with problem use and adverse school outcomes is vital to help inform public health recommendations and strategies to promote adolescent health. Fifth, the present study did not measure quality or content either, a limitation that could be addressed in future studies. Finally, the present study did not examine the role of the context of technology use on the associations of problematic technology use with academic performance and school connectedness, because the question on context of technology use was not asked to students. This is particularly important because previous studies have showed that the association between technology use and academic performance may differ between weekdays and weekends [60,61,62]. Future research that simultaneously measures problematic technology use and the specific context of technology use is needed to better understand its impact on the associations of problematic technology use with academic performance and school connectedness among adolescents.

## 5. Conclusions

In conclusion, this study shows that excessive technology use and problem technology use are common among adolescents. Females are more likely to report using technology for a longer duration and report problem technology use symptoms than their male counterparts. Results further indicate that excessive technology use and problem technology use are associated with lower academic performance and lower levels of school connectedness among male and female adolescents. These findings further support the current public health recommendation that children and youth should spend no more than 2 h per day in recreational screen time [6]. Our results also highlight the need to find ways to reduce time spent using technology as a possible means to promote positive school outcomes among secondary school students. There is a need for future investigations using prospective data to confirm directionality of relationships between problem technology use and academic performance and school connectedness among adolescents.

## Figures and Tables

**Table 1:** Descriptive characteristics of the study sample.

	Total Sample (*n* = 4837)	Females (*n* = 2802)	Males (*n* = 2035)	*p*-Value ^a^
Age (years)				
Mean (SD) (Min: 12, Max: 20)	15.9 (1.3)	15.9 (1.4)	15.9 (1.2)	0.295
Ethnoracial background				
White	51.4	52.4	50.3	0.300
Black	9.7	10.3	9.1	
East/South-East Asian	14.9	13.5	16.2	
South Asian	8.3	8.4	8.3	
Other	15.7	15.4	16.1	
Subjective socioeconomic status				
Mean (SD) (Min: 1, Max: 10)	6.8 (1.7)	6.8 (1.8)	6.9 (1.6)	0.139
Tobacco cigarette smoking				
Mean (SD) (Min: 1, Max: 10)	1.2 (0.8)	1.2 (0.8)	1.3 (0.8)	0.145
Alcohol consumption				
Mean (SD) (Min: 1, Max: 9)	2.7 (1.6)	2.8 (1.7)	2.7 (1.5)	0.297
Cannabis use				
Mean (SD) (Min: 1, Max: 7)	1.9 (1.8)	1.8 (1.7)	2.0 (1.8)	<0.001
Academic performance				
Mean (SD) (Min: 1, Max: 5)	3.7 (0.9)	3.9 (0.9)	3.5 (0.8)	<0.001
School connectedness				
Mean (SD) (Min: 3, Max: 12)	9.5 (1.8)	9.3 (1.9)	9.7 (1.6)	<0.001
Technology use				
2 h or less	29.3	25.0	33.6	<0.001
3 to 4 h	34.9	37.1	32.7	
5 h or more	35.8	37.9	33.8	
Problem technology use score				
Mean (SD) (Min: 0, Max: 24)	9.3 (4.7)	10.0 (5.0)	8.7 (4.2)	<0.001
Moderate-to-serious problem technology use				
No	81.4	77.6	85.1	<0.001
Yes	18.6	22.4	14.9	
Serious problem technology use				
No	97.1	96.2	98.1	0.005
Yes	2.9	3.8	1.9	

Data are shown as weighted column %, unless otherwise indicated. SD: standard deviation. ^a^ *p*-value of difference between females and males.

**Table 2:** Associations of time spent using technology with academic performance and school connectedness among adolescent females and males.

	Academic Performance				School Connectedness			
	Females (*n* = 2802)		Males (*n* = 2035)		Females (*n* = 2802)		Males (*n* = 2035)	
	OR	95% CI	OR	95% CI	OR	95% CI	OR	95% CI
**Model 1**								
Technology use								
2 h or less	1		1		1		1	
3 to 4 h	**0.71**	**0.52** **–** **0.95**	1.09	0.85–1.40	1.00	0.82–1.23	1.10	0.85–1.43
5 h or more	**0.44**	**0.33–0.59**	**0.67**	**0.51–0.89**	**0.70**	**0.56–0.88**	**0.64**	**0.50–0.82**
**Model 2**								
Technology use								
2 h or less	1		1		1		1	
3 to 4 h	**0.69**	**0.52–0.91**	1.13	0.88–1.45	1.00	0.81–1.23	1.11	0.85–1.45
5 h or more	**0.46**	**0.34–0.61**	**0.73**	**0.55–0.98**	**0.75**	**0.60–0.93**	**0.72**	**0.56–0.92**

OR: odds ratio; CI: confidence interval. Model 1: unadjusted. Model 2: adjusted for age, ethnoracial background, subjective socioeconomic status, tobacco cigarette smoking, alcohol consumption, and cannabis use. Bold values indicate statistical significance at *p* < 0.05.

**Table 3:** Associations of moderate-to-high symptoms of problem technology use with academic performance among adolescent females and males.

	Females (*n* = 2802)		Males (*n* = 2035)	
	OR	95% CI	OR	95% CI
**Model 1**				
Stayed on devices longer than intended	0.92	0.82–1.03	1.05	0.95–1.16
Neglected homework because spending more time on devices	**0.73**	**0.67–0.80**	**0.76**	**0.69–0.84**
Criticized by parents/friends about time spent on electronic devices	**0.88**	**0.80–0.96**	0.97	0.90–1.04
Lose sleep because you use devices late at night	**0.86**	**0.79–0.92**	**0.89**	**0.82–0.96**
Felt nervous when not using devices and felt relieved when using them	**0.77**	**0.71–0.85**	**0.79**	**0.72–0.87**
Choose to spend more time on electronic devices rather than go out with friends	**0.88**	**0.80–0.97**	**0.84**	**0.76–0.91**
Total score	**0.94**	**0.91–0.96**	**0.95**	**0.93–0.97**
Moderate-to-high symptoms of problem technology use				
No	1		1	
Yes	**0.63**	**0.50–0.80**	**0.66**	**0.52–0.83**
**Model 2**				
Stayed on devices longer than intended	0.92	0.81–1.03	1.08	0.98–1.19
Neglected homework because spending more time on devices	**0.74**	**0.68–0.81**	**0.79**	**0.72–0.87**
Criticized by parents/friends about time spent on electronic devices	**0.86**	**0.78–0.94**	0.98	0.91–1.05
Lose sleep because you use devices late at night	**0.87**	**0.81–0.94**	**0.92**	**0.85–0.99**
Felt nervous when not using devices and felt relieved when using them	**0.78**	**0.72–0.86**	**0.80**	**0.73–0.88**
Choose to spend more time on electronic devices rather than go out with friends	**0.89**	**0.82–0.97**	**0.84**	**0.77–0.92**
Total score	**0.94**	**0.91–0.96**	**0.96**	**0.94–0.98**
Moderate-to-high symptoms of problem technology use				
No	1		1	
Yes	**0.66**	**0.52–0.84**	**0.68**	**0.53–0.87**

OR: odds ratio; CI: confidence interval. Model 1: unadjusted. Model 2: adjusted for age, ethnoracial background, subjective socioeconomic status, tobacco cigarette smoking, alcohol consumption, and cannabis use. Bold values indicate statistical significance at *p* < 0.05.

**Table 4:** Associations of moderate-to-high symptoms of problem technology use with school connectedness among adolescent females and males.

	Females (*n* = 2802)		Males (*n* = 2035)	
	OR	95% CI	OR	95% CI
**Model 1**				
Stayed on devices longer than intended	0.91	0.83–1.00	0.88	0.81–0.96
Neglected homework because spending more time on devices	**0.84**	**0.76–0.94**	**0.78**	**0.69–0.87**
Criticized by parents/friends about time spent on electronic devices	**0.90**	**0.84–0.97**	**0.86**	**0.79–0.92**
Lose sleep because you use devices late at night	**0.87**	**0.82–0.93**	0.92	0.84–1.01
Felt nervous when not using devices and felt relieved when using them	**0.85**	**0.79–0.92**	**0.79**	**0.70–0.88**
Choose to spend more time on electronic devices rather than go out with friends	**0.70**	**0.63–0.77**	**0.70**	**0.63–0.77**
Total score	**0.94**	**0.92–0.96**	**0.93**	**0.91–0.95**
Moderate-to-high symptoms of problem technology use				
No	1		1	
Yes	**0.61**	**0.50** **–** **0.74**	**0.59**	**0.45–0.77**
**Model 2**				
Stayed on devices longer than intended	0.93	0.84–1.02	0.93	0.85–1.02
Neglected homework because spending more time on devices	**0.87**	**0.78–0.97**	**0.81**	**0.73–0.89**
Criticized by parents/friends about time spent on electronic devices	**0.89**	**0.83–0.96**	**0.88**	**0.81–0.96**
Lose sleep because you use devices late at night	**0.90**	**0.85–0.96**	0.97	0.89–1.06
Felt nervous when not using devices and felt relieved when using them	**0.87**	**0.81–0.94**	**0.83**	**0.74–0.93**
Choose to spend more time on electronic devices rather than go out with friends	**0.72**	**0.65–0.79**	**0.72**	**0.65–0.79**
Total score	**0.95**	**0.93–0.97**	**0.94**	**0.92–0.96**
Moderate-to-high symptoms of problem technology use				
No	1		1	
Yes	**0.63**	**0.51–0.78**	**0.65**	**0.50–0.86**

OR: odds ratio; CI: confidence interval. Model 1: unadjusted. Model 2: adjusted for age, ethnoracial background, subjective socioeconomic status, tobacco cigarette smoking, alcohol consumption, and cannabis use. Bold values indicate statistical significance at *p* < 0.05.

## Data Availability

Our data cannot be made available in the manuscript, the supplemental files or a public repositor due to the Centre for Addiction and Mental Health’s and The Ontario Public and Catholic School Board’s institutional Research Ethics Board agreements. Readers, however, may request the public data file underlying the findings of this study by contacting the Centre for Addiction and Mental Health at info@camh.ca.
